# Ischiospinal Dysostosis in a Child with Pierre-Robin Syndrome

**DOI:** 10.1155/2017/8263536

**Published:** 2017-04-03

**Authors:** Mahmoud Almasri, Waleed Kishta, Fahad H. Abduljabbar, Vincent Arlet, Neil Saran, Jean Oullet

**Affiliations:** ^1^McGill University, Shriners Hospital for Children, Montreal, QC, Canada; ^2^Department of Orthopedic Surgery, University of Ottawa, Ottawa, ON, Canada; ^3^Victoria Hospital, Western University, London, ON, Canada; ^4^Department of Orthopedic Surgery, King Abdulaziz University, Jeddah, Saudi Arabia; ^5^Department of Orthopedics, University of Pennsylvania, Philadelphia, PA, USA

## Abstract

Ischiospinal Dysostosis (ISD) is a complex and very rare medical entity. It is associated with kyphoscoliosis, dysplasia or aplasia of the ischial rami, segmental anomalies of the bony vertebrae, and peculiar facial morphologies. In this case report, we present a child with Ischiospinal Dysostosis and Pierre-Robin Syndrome. This case report is unique as we followed the patient for 13 years in which he had multiple spinal procedures to treat his kyphoscoliosis. In this paper, we elucidated the number of case reports with documented follow-up regarding spinal cord injury or other complications of ISD and its management.

## 1. Introduction 

Ischiospinal Dysostosis (ISD) is an extremely rare medical entity characterized by kyphoscoliosis, dysplasia or aplasia of the ischial rami, segmental anomalies of the bony vertebrae, and peculiar facial morphologies. To our knowledge, there exists only 24 reported cases in the literature that discuss individuals who display features of this syndrome [1–7]. While similarities exist between ISD and Cleidocranial Dysplasia (CCD), the vast majority of case studies highlight ISD as a related, yet uniquely separate disorder. ISD mimics CCD; however, as the clavicles are dysplastic in the latter, they remain normal in the former. Although ISD has been reported in individuals with a noncontributory family history, its inheritance appears to be autosomal dominant, as evidenced by several familial cases [1, 2].

We describe the case of a patient who suffers from Pierre-Robin Syndrome, but who also exhibits major features of ISD. Pierre-Robin Syndrome (PRS) is a congenital facial abnormality that consists of a cleft palate, small jaw (micrognathia), and retracted tongue (glossoptosis). Our case report is unique in that it documents the initial presentation and the medical and the surgical management of an ISD patient who, overall, has had over 13 years of follow-up. We document consequences of spinal manipulation surgery and the resulting complications from our procedures. We are also the first paper to elucidate the number of case reports with documented follow-up regarding spinal cord injury or other complications of ISD and its management.

## 2. Case Report 

We present a 15-year-old boy known for Pierre-Robin Syndrome. He is wheel-chair-bound, and suffers from global retardation and a significant autistic spectrum disorder. He is the third child of a mother who has ischiovertebral dysplasia and severe scoliosis. Pregnancy was 34 weeks. At birth, after a natural delivery, he weighed 4 lbs 15 oz and was found to have gross developmental delay and a congenital meningomyelocele.

**Figure 1:**
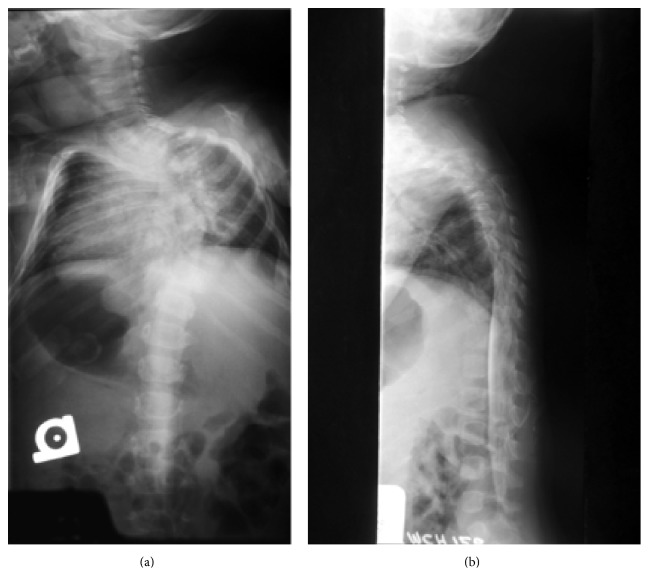
(a) PA (posteroanterior) view of the spine showing progressive cervicothoracic scoliosis. (b) Lateral view image of the spine showing severe kyphosis with rotatory subluxation at the level of T4-T5. The degree of subluxation almost gives the appearance of a congenital vertebral dislocation at the T4 on T5 junction.

**Figure 2:**
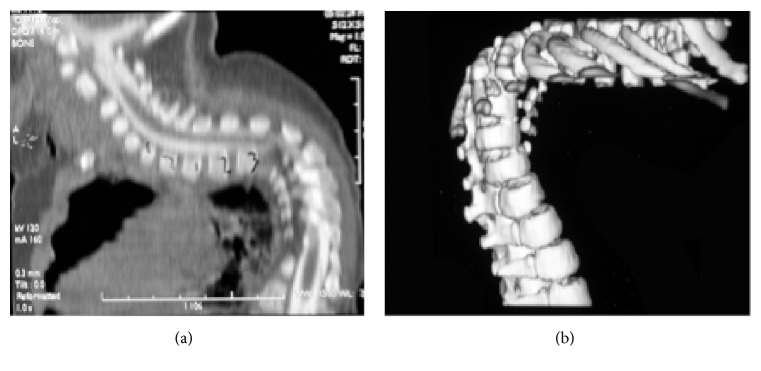
(a) Sagittal cut of a CT-scan (myelogram) of the spine showing kyphosis demonstrated in the plain radiographs of Figure 1. (b) Three-dimensional reconstruction of the thoracic spine showing the severity of kyphoscoliosis.

**Figure 3:**
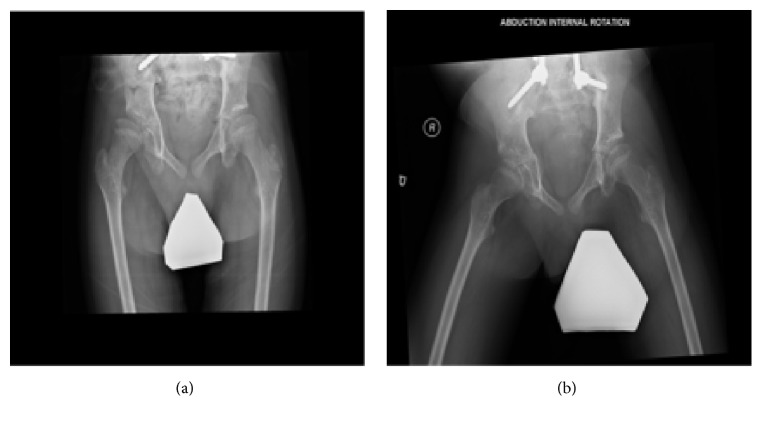
(a) AP radiograph of the pelvis and (b) abduction internal rotation views, both demonstrating bilateral dysplasia of the ischium.

He presented to the Shriner's hospital at 3 years of age with Spina Bifida, congenital vertebral dislocation, and severe congenital scoliosis (Figures 1 and 2). Skeletal survey at the time also demonstrated dysplasia of both his ischium and certain segments of his spinal column (Figures 1 and 3). Of note, the patient and his mother both have normal patellae bilaterally with normal extensor function which excludes ischiopatellar dysplasia.

At the age of 4, he underwent posterior spinal fusion from C6 to L1 utilizing USS (Universal Spinal System) and titanium rods. Two weeks later, he underwent anterior spinal fusion from C7 to T10 with a vertebral strut graft. This latter procedure was complicated with transient paraplegia, with loss of pain sensation and rectal tone. Although his baseline preoperative neurologic function had shown some long tract signs, both upper and lower limb strength and sensation were 4+ on 5. Therefore, his postoperative symptoms were suspected to be secondary to either direct spinal cord injury or vascular injury; however, the patient regained some function postoperatively after urgent decompressive laminectomies.

Following a likely iatrogenic spinal cord injury, there was residual spasticity of his lower extremities. Moreover, his neuromuscular scoliosis resulted in a neurogenic bowel and bladder. He was also left with indefinite asymmetric paraparesis with the left lower extremity affected more than the right.

His incontinence is managed with clean intermittent catheterization (CIC) and Ditropan. His kidneys were found normal with never any evidence of hydronephrosis nor cyst formation. He is fed by a gastrostomy tube and sleeps with a BiPAP/CPAP apparatus due to sleep apnea since infancy.

**Figure 4:**
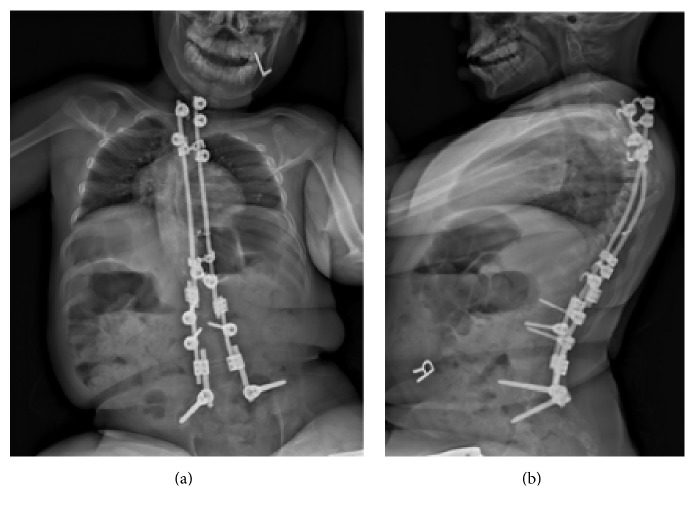
AP and lateral radiographs of the spine showing spinal instrumentation from the level of T1 down to the pelvis; the crank-shaft of his spine is also apparent here.

Subsequently, at 8 years of age, he sustained a pelvic obliquity and evidence of a wind-swept deformity at the wings of the iliac crest; this necessitated further spinal fusion down to the pelvis (Figure 4).

**Figure 5:**
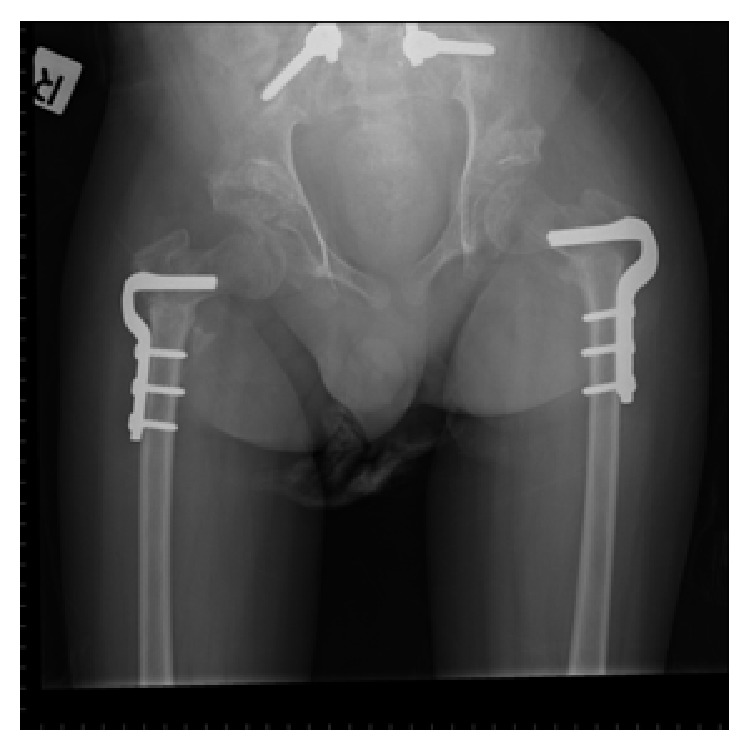
Postoperative AP radiograph of the pelvis after bilateral dega osteotomy and bilateral proximal femur varus derotational osteotomy.

From an orthopedic point of view, he developed a crank-shaft phenomenon of his spinal fusion and pseudarthrosis across his lumbosacral junction over the course of 8 years after surgery (Figure 4). His kyphosis, moreover, continued to worsen. He sustained bilateral hip subluxation requiring dega osteotomy plus a proximal femur varus derotation at the age of 15. On his last assessment by the orthopedics team, imaging showed no further signs of subluxation (Figure 5).

## 3. Discussion 

Patients with ISD may have facial, musculoskeletal, and nonmusculoskeletal manifestations, and the age at presentation can range from birth to adulthood. Causes for presentation were varied, but they revolved around seeking medical attention for childhood or adolescent scoliosis, delayed gross motor development in infants or children, congenital dislocation of the hips, chronic hip pain or back pain, club feet, lower limb sensory deficits, respiratory distress at birth, or cardiorespiratory failure in adulthood [1–7].

Nonmusculoskeletal manifestations of ISD extended to skin changes, renal abnormalities, and respiratory dysfunction. One ISD patient was reported to have increased skin laxity [2]. Although rare, bilateral polycystic kidneys were reported in 2 patients [4, 6]. These cysts were consistent with nephroblastomatosis in one of those cases [6] and had implied an increased risk of Wilm's tumor formation. The third case with renal involvement only showed a solitary cyst in her left kidney and with no evidence of renal dysfunction.

Secondary to severe kyphoscoliosis and short thoraces, changes in chest wall anatomy were imminent in many ISD patients; these often implied increased risk for respiratory dysfunction. Both one patient with Pierre-Robin Syndrome [2] and another reported case [3] had developed respiratory distress at birth. Another patient had died after developing cardiorespiratory failure at 33 years of age.

Defects to the ischial bones were found in all cases. Abnormalities were either confined to the ischial rami or may also have extended to the pubic bone. Issues were often due to nonossification, which naturally resulted in either hypoplastic or aplastic ischial rami. Incomplete fusion of the ischiopubic rami or absent superior and inferior pubic rami were also found. Nonossification of the sacrum was encountered in a patient who also had stipple-like ossification of his coccyx [3].

Major anomalies of the spine were detected in ISD. Firstly, 18 of the reported 24 cases involved some component of kyphosis and/or scoliosis to the thoracic and/or lumbar spine. In some patients the kyphoscoliosis was severe and rapidly progressing [1], while in others it was more stable over time [2, 5, 7]. Moreover, abnormalities at multiple vertebral segments often accompanied the kyphoscoliosis but could also be found independently. These vertebral irregularities were vast and included aberrant ossification centers of the cervicothoracic junction, irregularly ossified vertebrae, significantly widened or reduced interpedicular distances, butterfly vertebrae, hemivertebrae, block vertebrae, hypoplasia of the anterior vertebral bodies, widened or incompletely fused lumbar posterior arches, and nonossification of lower sacral segments.

Of importance, coexisting lumbar canal stenosis or narrowed lumbosacral canal was found in 4 cases [3], and each had led to neurological sequelae. By the age of 10, the first case demonstrated right lower leg paresis and incontinence with recurrent cystitis. Due to her concurrent lumbosacral hypoplasia and canal narrowing, she further sustained cauda equina syndrome and a neurogenic bladder. Likely due to decreased sensory feedback to the heel, she developed a nonhealing ulcer with a prolonged course. Despite lumbosacral laminectomy at ages 14 and 18, paresis of her lower legs worsened, and her left equinovarus deformity persisted. Nishimura's second case also suffered from a prolonged plantar ulcer and left equinovarus foot [3]. Mild flaccid paralysis and bilateral lower leg sensory deficits were described in their third case. Their fourth case did not seem to have neurological deficits despite lumbosacral narrowing. No surgeries were offered in any of their last 3 patients.

Spinal cord compression had also been witnessed by Cohen et al. in 4 out of their 8 patients; one of those patients also suffered from Pierre-Robin Syndrome [1]. In contrast to Nishimura's experience of neurological symptoms due to canal narrowing, Cohen attributed spinal cord compression due to severity of scoliosis. Interestingly, it was due to spinal manipulation during anaesthesia, before surgery, that symptoms manifested in their fifth case. In contrast, their last case only demonstrated evidence of cord damage after surgery. No comment was made on the timing of symptoms in their two other cord compression cases.

Surprisingly, lower cord diastematomyelia with spinal tethered cord syndrome (a.k.a. spinal dysraphism) in one ISD patient had no effect on either motor, sensory, or bladder function [5].

Overall, 9 from the 24 patients (37.5%) discussed here had developed neurological symptoms, with each of the 9 having never undergone spinal manipulation.

Surgical management of ISD was intermittently discussed in the literature, but logically, treatment was aimed at correcting the kyphoscoliosis or other bone and joint manifestations of the disorder. Cohen's group recommended circumferential spinal fusion at an early age (before 10 years of age) in order to circumvent future morbidity or mortality from spinal cord compression or deterioration of pulmonary function. They also discussed the use of skeletal traction and discussed combining posterior fusion with concave strut stabilization anteriorly, when the patient is older than 10 [1]. The only other mention of spinal surgery was already noted above and was that of laminectomies to relieve spinal cord compression [3]. Bilateral arthrodesis for clubfeet was performed in another case [3].

## 4. Conclusion 

Our study is the first of its kind to provide greater than 13 years of follow-up to spinal manipulation surgery and its outcomes on a case of ISD. It is only the second to demonstrate a possible relationship between Pierre-Robin Syndrome and ISD.

Our patient demonstrates clinical features with considerable overlap to others with the same disorder. Moreover, the natural history of his disease and the spinal manifestations seems to follow the majority of other ISD cases who have never even underwent spine surgery. Although, in our case, iatrogenic spinal cord injury seemed responsible for the subsequent neurogenic symptoms, we cannot conclude that these sequelae were not intrinsic to the syndrome itself.
